# A myco-management problem: improving utilization of fungal and mycobacterial smear and culture

**DOI:** 10.1128/jcm.00030-26

**Published:** 2026-06-09

**Authors:** Sarah M. Schrader, Vamsi Thiriveedhi, John A. Branda, Sarah E. Turbett, Erik H. Klontz

**Affiliations:** 1 Department of Pathology, Massachusetts General Hospital 272105, , Boston, Massachusetts, USA; 2 Department of Pathology, Brigham and Women’s Hospital 550233, , Boston, Massachusetts, USA; 3 Harvard Medical School 1811 , Boston, Massachusetts, USA; 4 Division of Infectious Diseases, Massachusetts General Hospital 2348, , Boston, Massachusetts, USA; University of Utah, Salt Lake City, Utah, USA

**Keywords:** test utilization, mycobacterial diagnostics, fungal diagnostics

## Abstract

Fungal and mycobacterial smear and culture are often inappropriately ordered, straining clinical laboratory resources and risking patient harm from false-positive results. To identify opportunities for optimization, we assessed utilization and yield of fungal and mycobacterial smear and culture over five years at a large academic medical center in an area of low tuberculosis prevalence. Positive rates were generally lower for non-respiratory specimens compared to respiratory specimens, prompting us to focus on collection method (swab vs non-swab) and location (operating room [OR] vs non-OR) for non-respiratory specimens. For non-respiratory fungal smear and culture, swabs demonstrated poor recovery of mold in culture, and positive smear results from swabs rarely affected clinical management (6/3,611 cases [0.17%]). Positive rates were significantly lower among OR specimens. For mycobacterial smear and culture, positive rates were exceedingly low for non-respiratory specimens (smear: 70/23,661 [0.3%]; culture: 278/23,918 [1.2%]). Swabs were inferior for mycobacterial culture, and positive smear results from swabs rarely impacted clinical management (3/7,398 cases [0.04%]). Among patients with at least one non-respiratory mycobacterial smear or culture over a two-year period, the smear or culture was diagnostic in only 32/4,572 (0.70%), 31 (96.9%) of whom had risk factors for mycobacterial infection. The smear result changed clinical management in only 5/4,497 cases (0.11%). Our results reveal considerable overutilization of fungal and mycobacterial smear and culture for non-respiratory specimens and confirm the low yield of non-respiratory swabs for mold and mycobacterial detection. These data inform strategies to optimize fungal and mycobacterial diagnostic testing in clinical laboratories.

**IMPORTANCE:**

Fungal and mycobacterial infections are associated with specific risk factors. However, diagnostic studies for fungal and mycobacterial infections, including fungal and mycobacterial smear and culture, are often inappropriately ordered in clinical scenarios where the probability of infection is low. Due to the manual nature of these tests, high volumes of unnecessary testing strain limited clinical laboratory resources. In addition, patients can be harmed by false-positive results. In this study, we analyzed the results of fungal and mycobacterial smear and culture performed over five years at a large academic medical center in a location with low tuberculosis prevalence to identify strategies to reduce unnecessary testing. Implementation of these strategies could allow clinical laboratories to optimize fungal and mycobacterial diagnostic testing and more efficiently use limited clinical laboratory resources without compromising patient care.

## INTRODUCTION

Although many fungi and some mycobacteria can be detected using routine Gram stain and bacterial culture, most clinical microbiology laboratories employ specialized staining and culture techniques to improve detection (1–4). Many of these techniques require multiple media types and stains, and cultures require complex processing steps and prolonged incubation times. While these procedures are part of standard clinical laboratory workflows, they are labor intensive, and optimal performance requires specialized training, maintenance of competency and proficiency, and biosafety precautions. Additionally, the complexities inherent to specimen processing and result interpretation raise the potential for inaccurate results if testing is not performed correctly. Thus, strategies to limit unnecessary fungal and mycobacterial smear and culture testing are needed to preserve limited laboratory resources and prevent erroneous results that could hinder patient care.

Fungal and mycobacterial smears and cultures are often inappropriately ordered when the probability of infection is low (5–11). As these infections are frequently associated with specific risk factors, including severe illness, immunocompromising conditions, and select epidemiological exposures, targeting this testing to individuals with such risk factors could theoretically improve clinical yield while simultaneously reducing laboratory impact. In addition, providers often use suboptimal specimen collection methods, such as swabbing. While convenient, swabs are generally considered to provide low-quality specimens for most microbiologic cultures due to their limited ability to capture and release adequate quantities of microorganisms and are especially discouraged for fungal and mycobacterial studies (12–15).

Characterization of clinical scenarios in which a specimen is unlikely to yield actionable results could inform interventions to optimize utilization of fungal and mycobacterial smear and culture. Here, we examine fungal and mycobacterial smear- and culture-positive rates at a large academic medical center in an area with low tuberculosis prevalence over a five-year period to identify opportunities to improve use. We focus on specimen source (respiratory vs non-respiratory), collection method (swab vs non-swab), collection location (operating room [OR] vs non-OR), presence or absence of risk factors for infection, and clinical impact of positive results to help inform stewardship strategies that could be applied in the clinical setting.

## MATERIALS AND METHODS

### Data collection

We retrospectively queried the Mass General Brigham (MGB) electronic health record (Epic from Epic Systems, Verona, WI, USA) for all fungal smear, fungal culture, mycobacterial smear, and mycobacterial culture orders placed as part of routine clinical care between 1 January 2020 and 31 December 2024 for which testing was performed at the Massachusetts General Hospital (MGH) Clinical Microbiology Laboratory, which is CLIA-certified for high-complexity testing and services MGH and its affiliated outpatient centers (along with Salem Hospital, an MGB-affiliated community hospital, for mycobacterial smear and culture). The tested population includes oncology, solid organ and bone marrow transplant, and cystic fibrosis patients. We excluded orders for which the specimen was refused or not collected and orders that were credited, canceled, or (for mycobacterial culture) reported as unsatisfactory due to overgrowth of contaminants. Specimen processing and result reporting methods for fungal smear and culture and mycobacterial smear and culture are detailed in the supplemental material.

### Specimen classification

To investigate the influence of specimen type (respiratory vs non-respiratory, plus vaginal for fungal smear/culture), collection method (swab vs non-swab), and collection location (OR vs non-OR) on positive rates for fungal and mycobacterial smear and culture, we used keyword searches from the specimen description to classify each specimen as (i) vaginal or non-vaginal, (ii) respiratory or non-respiratory, (iii) swab or non-swab, and (iv) collected inside or outside the OR. Respiratory specimen definitions are described in the supplemental material. The standard swab used in our hospital during this period was the BBL CultureSwab (Becton, Dickinson and Company, Franklin Lakes, NJ, USA). We then used keyword searches in the result text to classify the result as positive or negative. For fungal studies, we characterized the result as indicative of yeast, mold, or both. Specimen classifications were performed in an automated fashion using Python scripts. Keywords used for specimen classification are described in the supplemental material. A random subset of 50 specimens for each test (fungal smear, fungal culture, mycobacterial smear, and mycobacterial culture) was manually reviewed to confirm accurate classification.

### Analysis of fungal and mycobacterial smear-positive cases from swabs

To understand how often positive fungal and mycobacterial smear results from swabs were clinically meaningful, we selected as a representative set all non-vaginal swab specimens from the five-year analysis period for which fungal or mycobacterial smear was positive, since the relatively small number of specimens permitted detailed chart review of each case. Two independent physicians reviewed each chart to determine whether the results were clinically impactful. A result was considered clinically impactful if (i) no concurrent non-swab specimen had a smear or Gram stain with the same result; (ii) the result was considered to represent a true pathogen based on documentation in the clinical notes; and (iii) the result prompted a change in clinical management (see supplemental material for additional details). Discrepancies were resolved by a third physician.

### Analysis of mycobacterial smear- and/or culture-positive, non-respiratory cases

Focusing on non-respiratory specimens, we further characterized ordering practices, patient characteristics, and clinical utility for patients with at least one positive mycobacterial smear and/or culture from a non-respiratory specimen collected between 1 January 2021 and 31 December 2022 as a representative sample. Two independent physicians performed detailed chart review to determine whether risk factors for mycobacterial infection were present and whether the results were clinically impactful (see supplemental material). Discrepancies were resolved by a third physician. To estimate the clinical effect of limiting the number of non-respiratory specimens accepted for mycobacterial culture, we selected patients for whom mycobacterial culture had provided a microbiologic diagnosis and used the total number of specimens and number of culture-positive specimens to determine the probability of at least one positive culture if the number of non-respiratory mycobacterial cultures had been limited to one, two, or three (see supplemental material). To estimate the laboratory workload reduction under different non-respiratory specimen limits, we calculated the number of mycobacterial smears and cultures that would have been performed based on the number of unique patients who received a mycobacterial smear and culture in 2021–2022.

### Statistical analysis

Proportions were compared using the chi-square test (performed in Python with the statsmodels.stats.proportion.proportions_chisquare function) with *P *values adjusted for multiple comparisons using the Bonferroni correction. Statistical significance was defined as a *P* value of <0.05.

## RESULTS

### Positive rates for fungal smear and culture

We included a total of 27,646 specimens processed for fungal smear and 65,877 specimens processed for fungal culture in our analysis. The overall positive rates were 8.2% (2,268/27,646) for smear and 19.0% (12,532/65,877) for culture.

#### Vaginal swabs

Among vaginal swabs, 15.8% (181/1,148) were fungal smear positive, and 25.6% (2,109/8,230) were fungal culture positive, with culture detecting fungi significantly more often than smear (25.6% vs 15.8%, *P* < 0.0001). Yeast alone was found in 97.8% (177/181) of positive fungal smears, with the remaining 2.2% (4/181) reported as unspecified fungal elements. Yeast alone grew in 99.8% (2,105/2,109) of fungal cultures; mold alone grew in 0.1% (3/2,109); and both yeast and mold grew in 0.05% (1/2,109).

#### Respiratory vs non-respiratory specimens

**Fig 1:**
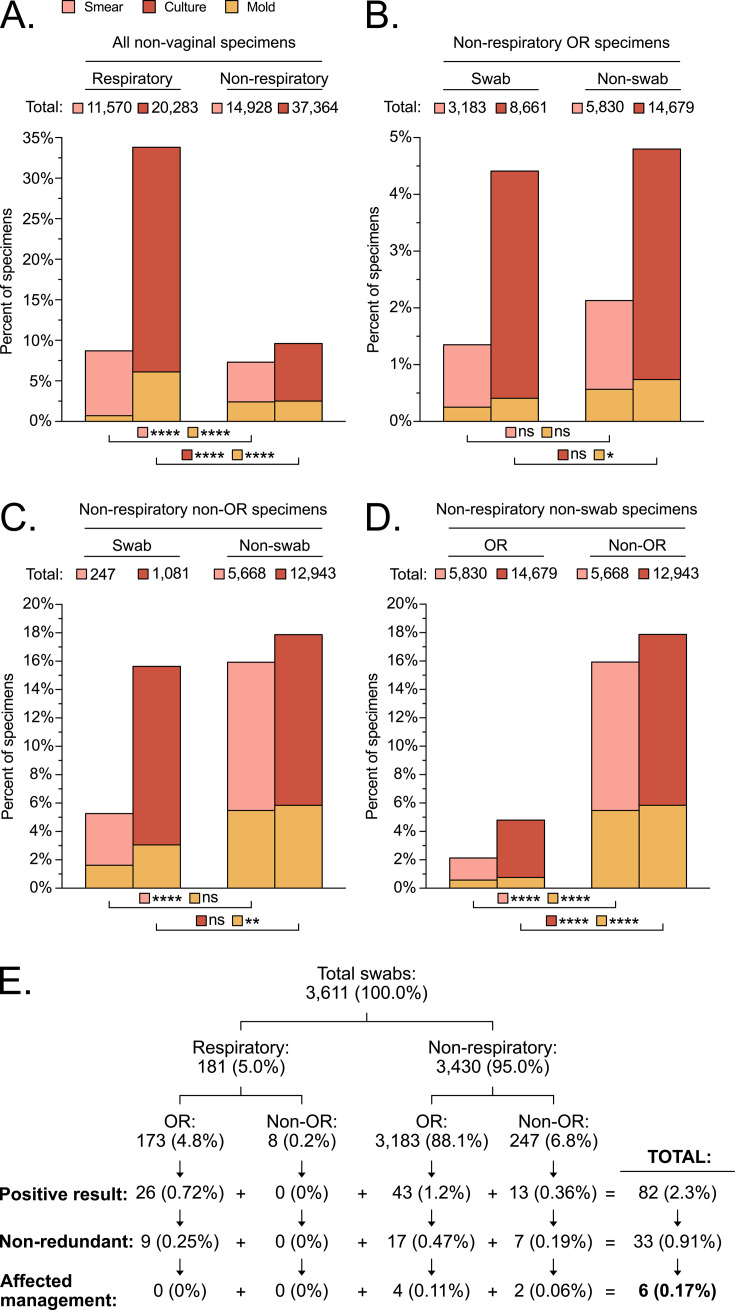
Fungal smear- and culture-positive rates and clinical utility of fungal smears performed on swabs. (**A**) Comparison of positive rates for non-vaginal respiratory vs non-respiratory specimens. (**B and C**) Comparison of positive rates for non-respiratory swab vs non-swab specimens collected in the operating room (OR) (**B**) and outside the OR (**C**). (**D**) Comparison of positive rates for non-respiratory, non-swab specimens collected in the OR vs outside the OR. Numbers under the subheadings at the top of each graph indicate the total number of tested specimens in that category for each test. Bars represent the percentage of tested specimens with a positive result. Statistical significance is indicated below the bars. *P* values for panels A–D were calculated using the chi-square test and were adjusted for multiple comparisons using the Bonferroni correction (based on 17 tests). ns, not significant; *, *P* < 0.05; **, *P* < 0.01; ****, *P* < 0.0001. (**E**) Clinical utility of fungal smears performed on swabs. “Non-redundant” indicates that no concurrently collected specimen yielded the same result.

Among respiratory specimens, 8.7% (1,004/11,570) were fungal smear positive, and 33.8% (6,854/20,283) were fungal culture positive. Compared to respiratory specimens, positive rates for non-vaginal, non-respiratory specimens were significantly lower: 7.3% (1,083/14,928) were fungal smear positive (*P* < 0.001), and 9.6% (3,569/37,364) were fungal culture positive (*P* < 0.0001) (Fig. 1A). Fungal smear results indicating the presence of mold were uncommon among both respiratory and non-respiratory specimens, with higher rates seen in non-respiratory specimens (2.4% [355/14,928] for non-respiratory vs 0.7% [78/11,570] for respiratory; *P* < 0.0001). Despite the higher fungal smear-positive rate for mold, fungal cultures from non-respiratory specimens yielded mold at a lower rate compared to respiratory specimens (2.5% [937/37,364] for non-respiratory vs 6.1% [1,246/20,283] for respiratory; *P* < 0.0001) (Fig. 1A).

#### Influence of collection method on positive rates for non-respiratory specimens collected in the OR

Given the lower fungal smear- and culture-positive rates for non-respiratory specimens, we further examined the influence of collection method (swab vs non-swab) and collection location (OR vs non-OR) on positive rates for this group of specimens. For non-respiratory, OR-collected specimens, overall fungal smear- and culture-positive rates were 1.9% (167/9,013) and 4.7% (1,087/23,340), with 35.3% (3,183/9,013) of specimens for smear and 37.1% (8,661/23,340) of specimens for culture collected on swabs. Fungal smear- and culture-positive rates were similarly low for both swab and non-swab specimens (fungal smear: 1.4% [43/3,183] for swabs vs 2.1% [124/5,830] for non-swabs, *P* ≥ 0.05; fungal culture: 4.4% [382/8,661] for swabs vs 4.8% [705/14,679] for non-swabs, *P* ≥ 0.05) with a trend toward lower detection of true hyphae on smear from swabs, although the difference did not reach statistical significance (0.3% [8/3,183] for swabs vs 0.6% [33/5,830] for non-swabs; *P* ≥ 0.05) (Fig. 1B). Although mold detection rates in fungal culture were universally low, cultures from swab specimens yielded mold at a significantly lower rate than cultures from non-swab specimens (0.4% [37/8,661] for swabs vs 0.8% [111/14,679] for non-swabs; *P* < 0.05) ( Fig. 1B). Most culture-positive specimens were from tissue sources (77.7% of swabs and 80.7% of non-swab specimens).

#### Influence of collection method on positive rates for non-respiratory specimens collected outside the OR

For non-respiratory, non-OR specimens, overall fungal smear- and culture-positive rates were 15.5% (916/5,915) and 17.7% (2,482/14,024), with 4.2% (247/5,915) of specimens for smear and 7.7% (1,081/14,024) of specimens for culture collected on swabs. The fungal smear-positive rate was significantly lower for swab specimens (5.3% [13/247] for swabs vs 15.9% [903/5,668] for non-swabs; *P* < 0.0001) (Fig. 1C). Detection of true hyphae was also lower for swab specimens, but the difference did not reach statistical significance (1.6% [4/247] for swabs vs 5.5% [310/5,668] for non-swabs; *P* ≥ 0.05). Although overall fungal culture-positive rates did not differ significantly between the two specimen types (15.6% [169/1,081] for swabs vs 17.9% [2,313/12,943] for non-swabs; *P* ≥ 0.05), cultures from swab specimens yielded mold at a significantly lower rate than cultures from non-swab specimens (3.1% [33/1,081] for swabs vs 5.8% [756/12,943] for non-swabs; *P* < 0.01) (Fig. 1C). The distribution of specimen sources for culture-positive non-swab vs swab specimens differed: most culture-positive non-swab specimens came from nails (31.3%), esophageal brushings (23.7%), tissue (16.2%), nose/mouth/throat (7.8%), skin (4.7%), or peritoneal fluid (2.6%), while most culture-positive swab specimens came from nose/mouth/throat (36.7%), tissue (22.5%), ear (14.2%), or skin (11.8%).

#### Influence of collection location on positive rates for non-respiratory specimens

Among non-respiratory, non-swab specimens, those collected outside the OR had significantly higher fungal smear- and culture-positive rates than those collected in the OR (fungal smear: 15.9% [903/5,668] for non-OR vs 2.1% [124/5,830] for OR, *P* < 0.0001; fungal culture: 17.9% [2,313/12,943] for non-OR vs 4.8% [705/14,679] for OR, *P* < 0.0001) (Fig. 1D). Both fungal smear and culture detected mold more frequently for non-OR specimens (fungal smear: 5.5% [310/5,668] for non-OR vs 0.6% [124/5,830] for OR, *P* < 0.0001; fungal culture: 5.8% [756/12,943] for non-OR vs 0.8% [111/14,679] for OR, *P* < 0.0001).

### Clinical utility of positive fungal smear results from swabs

Of the 82 swabs that had positive fungal smears, 33 (40.2%) provided non-redundant information (i.e., no concurrently collected specimen had a fungal smear or Gram stain with the same findings) (Fig. 1E). Of those, only six (18.2%) changed clinical management, representing 0.2% of the 3,611 total swabs processed for fungal smear. In five of the six cases, the smear showed budding yeast, and antifungal therapy was started. Concomitant cultures were positive in only three of five cases. In the sixth case, septated hyphae were detected on smear, prompting surgical debridement. Concomitant culture grew a *Penicillium* species. See Table S1 for additional details.

### Positive rates for mycobacterial smear and culture

We included a total of 48,911 specimens processed for mycobacterial smear and 48,874 specimens processed for mycobacterial culture in our analysis. The overall positive rates were 1.8% (888/48,911) for smear and 5.7% (2,767/48,874) for culture.

#### Respiratory vs non-respiratory specimens

**Fig 2:**
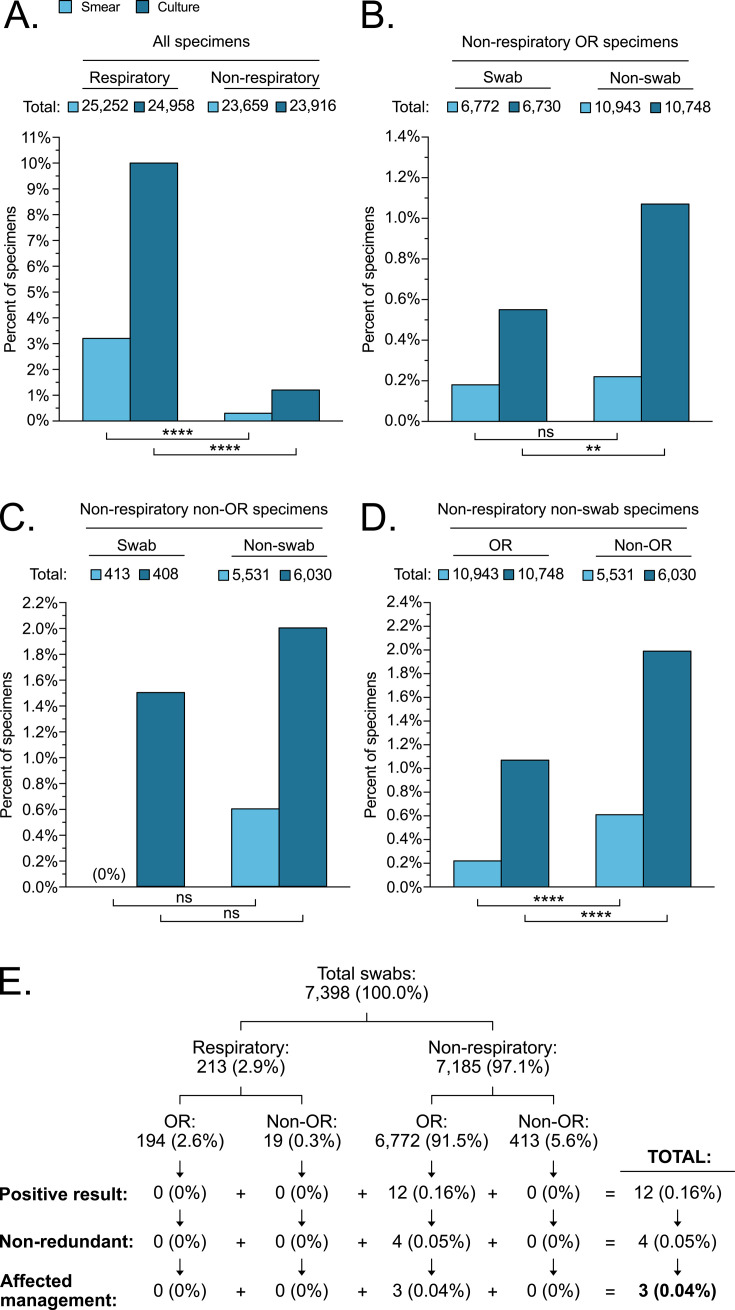
Mycobacterial smear- and culture-positive rates and clinical utility of mycobacterial smears performed on swabs. (**A**) Comparison of positive rates for respiratory vs non-respiratory specimens. (**B and C**) Comparison of positive rates for non-respiratory swab vs non-swab specimens collected in the operating room (OR) (**B**) and outside the OR (**C**). (**D**) Comparison of positive rates for non-respiratory, non-swab specimens collected in the OR vs outside the OR. Numbers under the subheadings at the top of each graph indicate the total number of tested specimens in that category for each test. Bars represent the percentage of tested specimens with a positive result. Statistical significance is indicated below the bars. *P* values were calculated using the chi-square test and were adjusted for multiple comparisons using the Bonferroni correction (based on eight tests). ns, not significant; **, *P* < 0.01; ****, *P* < 0.0001. (**E**) Clinical utility of mycobacterial smears performed on swabs. “Non-redundant” indicates that no concurrently collected specimen yielded the same result.

Among respiratory specimens, 3.2% (818/25,252) were mycobacterial smear positive, and 10.0% (2,489/24,958) were mycobacterial culture positive. As with fungal smear and culture, positive rates for non-respiratory specimens were significantly lower: 0.3% (70/23,659) were mycobacterial smear positive (*P* < 0.0001), and 1.2% (278/23,916) were mycobacterial culture positive (*P* < 0.0001) (Fig. 2A).

#### Influence of collection method on positive rates for non-respiratory specimens collected in the OR

Given the lower mycobacterial smear- and culture-positive rates for non-respiratory specimens and the inherent difficulty in implementing mycobacterial diagnostic stewardship strategies for respiratory specimens due to the reliance of tuberculosis rule-out protocols on these tests, we further examined the influence of collection location (OR vs non-OR) and collection method (swab vs non-swab) on positive rates for non-respiratory specimens. For non-respiratory, OR-collected specimens, overall mycobacterial smear- and culture-positive rates were 0.2% (36/17,715) and 0.9% (152/17,478), with 38.2% (6,772/17,715) of specimens for smear and 38.5% (6,730/17,478) of specimens for culture collected on swabs. Mycobacterial smear-positive rates were equally low for swab and non-swab specimens (0.2% [12/6,772] for swabs vs 0.2% [24/10,943] for non-swabs; *P* ≥ 0.05), but the mycobacterial culture-positive rate was significantly lower for swab specimens (0.6% [37/6,730] for swabs vs 1.1% [115/10,748] for non-swabs; *P* < 0.01) (Fig. 2B). Most culture-positive specimens were from tissue sources (97.3% of swabs and 95.7% of non-swab specimens).

#### Influence of collection method on positive rates for non-respiratory specimens collected outside the OR

For non-respiratory, non-OR specimens, overall mycobacterial smear- and culture-positive rates were 0.6% (34/5,944) and 2.0% (126/6,438), with 6.9% (413/5,944) of specimens for smear and 6.3% (408/6,438) of specimens for culture collected on swabs. Mycobacterial smear- and culture-positive rates were lower for swab specimens, but the differences did not reach statistical significance (mycobacterial smear: 0% [0/413] for swabs vs 0.6% (34/5,531) for non-swabs, *P* ≥ 0.05; mycobacterial culture: 1.5% [6/408] for swabs vs 2.0% [120/6,030] for non-swabs, *P* ≥ 0.05) (Fig. 2C). The distribution of specimen sources for culture-positive non-swab vs swab specimens differed: 83.3% of swabs came from tissue sources, while non-swabs came primarily from tissue sources (46.7%), urine (25.0%), and other fluids (15.8%).

#### Influence of collection location on positive rates for non-respiratory specimens

Among non-respiratory, non-swab specimens, those collected outside the OR had significantly higher mycobacterial smear- and culture-positive rates than those collected in the OR (mycobacterial smear: 0.6% [34/5,531] for non-OR vs 0.2% [24/10,943] for OR, *P* < 0.001; mycobacterial culture: 2.0% [120/6,030] for non-OR vs 1.1% [115/10,748] for OR, *P* < 0.0001) (Fig. 2D).

### Clinical utility of positive mycobacterial smear results for non-respiratory specimens received on swabs

All 12 mycobacterial smear-positive swabs were collected in the OR from non-respiratory specimens (Fig. 2E). Four provided non-redundant information. Only 3/7,398 (0.04%) changed clinical management. In one case, antimycobacterial therapy was started five days earlier than would have been possible without the smear. In the second, empiric antibiotic therapy was discontinued two days earlier than would have otherwise been possible. In the third case, the smear was ultimately considered to represent a false positive and resulted in a negative patient impact. See Table S2 for additional details.

### Clinical utility of positive mycobacterial smear and culture results for non-respiratory specimens

In 2021–2022, our mycobacteriology laboratory performed 20,975 smears on specimens from 8,506 unique patients and 20,851 cultures on specimens from 8,507 unique patients. Of these, 11,030/20,975 (52.6%) smears from 4,497/8,506 (52%) unique patients and 11,057/20.851 (53.0%) cultures from 4,539/8,507 (53.4%) were performed on non-respiratory specimens. A total of 4,572 unique patients had at least one mycobacterial smear and/or culture performed. The average numbers of non-respiratory smears and cultures per patient were 2.5 (SD ±2.6, range: 1–42) and 2.4 (SD ±2.6, range: 1–38), respectively.

**Fig 3:**
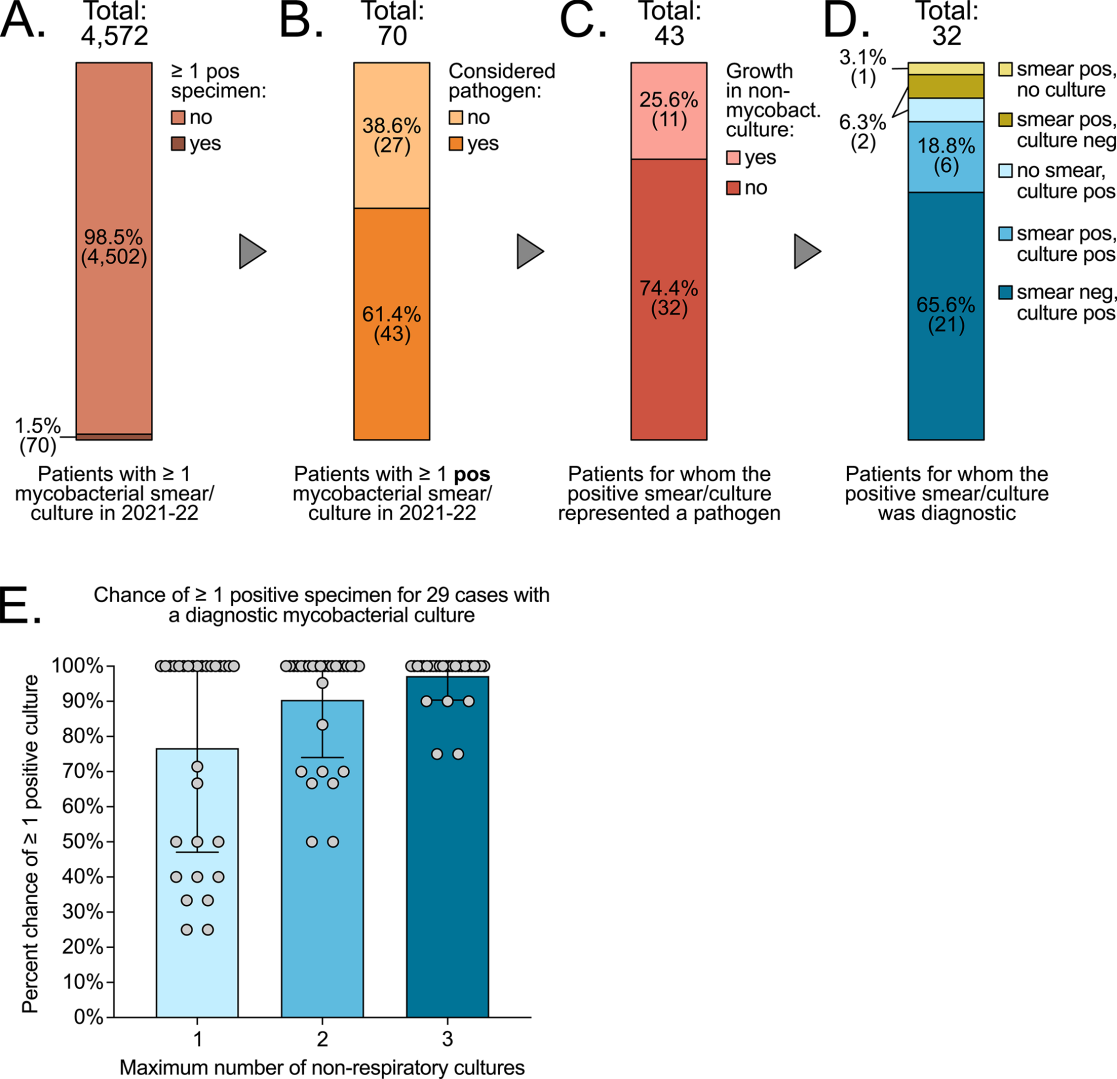
Characteristics of cases with at least one positive mycobacterial smear and/or culture result on a non-respiratory specimen in 2021–2022. (**A**) Percentage of patients with at least one non-respiratory mycobacterial smear and/or culture performed in 2021–2022 who had at least one positive result. (**B**) Percentage of patients with at least one positive result for whom the result was considered to represent a pathogen. (**C**) Percentage of patients with a presumed pathogen identified on mycobacterial smear and/or culture for whom the organism did not grow in non-mycobacterial culture. (**D**) Breakdown of smear vs culture results for patients in whom mycobacterial smear or culture alone provided the diagnosis. For all panels, each section of each bar is labeled with the percentage of patients it represents with the number of patients in parentheses. The total number of patients is indicated at the top of each bar. (**E**) Percent chance of at least one positive culture for the 29 cases with a diagnostic non-respiratory mycobacterial culture result, given a maximum number of non-respiratory cultures of one, two, or three. The bar represents the average percent chance of at least one positive culture across all cases, and the error bar represents ± standard deviation. Gray dots represent percent chance of at least one positive culture for individual cases. See supplemental material for more details on the calculations.

In total, only 70/4,572 patients (1.5%) had at least one positive smear and/or culture from a non-respiratory specimen (Fig. 3A), with the result considered to represent a pathogen in 43/70 (61.4%) patients (Fig. 3B). In 11/43 (25.6%) cases, the pathogen grew in both mycobacterial and a concurrent non-mycobacterial culture (Fig. 3C). This left 32/43 (74.4%) cases for which either the mycobacterial smear or culture provided a diagnosis (Fig. 3D). Of these patients, 31/32 (96.9%) had risk factors for mycobacterial infection. Twenty-one of 32 (65.6%) cases were smear negative and culture positive, and in 2/32 (6.3%) cases, only culture was performed per laboratory protocol (urine specimens). The remaining 9/32 (28.1%) cases were smear positive, including one for which culture was not performed per laboratory protocol (stool specimen), two for which concomitant culture was negative, and six for which concomitant culture was also positive. Overall, the smear result directly changed management in only 5/4,497 (0.1%) patients who had at least one non-respiratory mycobacterial smear performed. Details of these cases can be found in Table S3.

### Effect of mycobacterial culture limits on case detection rate

For the 29 mycobacterial culture-positive patients, we examined the effect of limiting the number of non-respiratory specimens accepted for mycobacterial culture to one, two, or three on the likelihood of each patient being diagnosed. For a limit of one, two, or three non-respiratory cultures, the average chance of a given case being diagnosed was 76.7% (SD ±29.7%), 90.4% (SD ±16.4%), and 97.2% (SD ±6.9%) ( Fig. 3E). On average, ~7 (7/29 = 24.1%), ~3 (3/29 = 10.3%), and ~1 (1/29 = 3.4%) cases would be missed at each limit.

### Effect of non-respiratory specimen limits on laboratory workload

Based on the total 2021–2022 mycobacterial smear and culture volumes for all specimen types (see supplemental material), we estimated that the total smear workload would be reduced by 52.6%, 31.1%, and 20.7% if zero, one, or two smears per patient were permitted from non-respiratory specimens. The total culture workload would be reduced by 31.3%, 20.7%, and 14.1% if one, two, or three non-respiratory cultures were permitted per patient.

## DISCUSSION

Here, we analyzed fungal and mycobacterial smear and culture data from five years of testing at a large academic medical center. We found all tests to be overutilized, with positive rates generally lower among non-respiratory specimens, specimens sent from the OR, and swab-collected specimens. Our findings suggest several strategies for improving utilization of these resource-intensive tests.

First, swabs are suboptimal for the collection of specimens for fungal and mycobacterial diagnostics. For fungal diagnostics, we observed a trend toward lower detection of mold on fungal smears (which likely did not achieve statistical significance due to small numerators) and significantly lower rates of mold growth from non-respiratory specimens collected on swabs. While differing specimen sources for swab vs non-swab specimens collected outside the OR could explain some of this difference, specimen sources for OR-collected specimens were similar for swab and non-swab specimens. For mycobacterial diagnostics, although mycobacterial smear-positive rates were comparably low for both swab-collected and non-swab-collected non-respiratory specimens, among OR-collected specimens, the culture-positive rate for non-respiratory swabs was almost 50% lower than that of non-respiratory, non-swab specimens. In addition, even when swab smears were positive, only a very small proportion—0.17% of all swabs submitted for fungal smear and 0.04% of all swabs submitted for mycobacterial smear—resulted in changes to clinical management. Most changes were modest (e.g., slightly earlier initiation of antimicrobial therapy), and in one case (case 3 in Table S2), the positive mycobacterial smear was ultimately determined to not represent true infection and resulted in harm to the patient (unnecessary debridement). Thus, fungal and mycobacterial smears from swabs rarely provided a clinical benefit. These findings support recommendations against swab collection for fungal and mycobacterial diagnostics (12–14). Since most swabs were collected in the OR, strategies to educate surgical teams and/or limit availability of swabs in the OR setting could help reduce swab use for specimen collection.

Second, for fungal diagnostics, non-respiratory specimens collected in the OR had lower rates of fungal smear and culture positivity than those collected outside the OR, both overall and for mold. Lower contamination rates of OR-derived specimens with oral, skin, and mucosal flora, which can include both yeast and mold (16), could underlie some of these differences. However, our specimen source comparison also suggests that the differential culture-positive rates might be driven by more syndromic-based ordering for specimens collected outside the OR, which primarily came from common sites of yeast or dermatophyte infection, including nails, esophageal brushings, nose/mouth/throat samples, and skin. In contrast, most culture-positive OR specimens were tissues or bone, suggesting that OR ordering practices might be motivated less by clinical suspicion for a fungal infection and more by a desire to ensure all diagnostic bases have been covered. Clinical decision tools that query risk factors for invasive yeast or mold infection (see supplemental material) could help reduce unnecessary testing, especially in the OR setting. In support of this, clinical scores that have been developed to evaluate the risk of invasive candidiasis in critically ill patients (17) and invasive mold infection in patients with hematologic malignancies (18) have high negative predictive values.

Third, based on our analysis of positive fungal and mycobacterial smears from swabs, 60% of positive fungal smears and 75% of positive mycobacterial smears provided information that was redundant with results of concurrently collected specimens. This finding suggests that in many cases, more specimens than necessary are sent from the same procedure. Although outside the scope of the current study, further analysis to determine the clinical scenarios in which multiple concurrent specimens are most often sent could help inform strategies for reducing unnecessary test volume.

For mycobacterial smear and culture, non-respiratory specimens had very low overall positive rates that were approximately 10-fold lower than the corresponding positive rates for respiratory specimens. This finding suggests a high degree of mycobacterial diagnostic overutilization for non-respiratory specimens and highlights a need to develop strategies to improve yield. Our review of cases with at least one positive mycobacterial smear and/or culture from a non-respiratory specimen suggests several potential strategies.

First, mycobacterial smear was rarely clinically meaningful for non-respiratory specimens, with positive results leading to a change in clinical management in only 0.1% of cases. This finding suggests that elimination of routine mycobacterial smears on non-respiratory specimens could significantly reduce smear volume with minimal impact on patient care. A dedicated approval process for mycobacterial smear from non-respiratory specimens could be implemented for select cases where clinical suspicion for mycobacterial infection is high and a positive result would be clinically actionable, further minimizing any impact on patient care with relatively little cost to the clinical laboratory.

Second, our data suggest that limiting the number of non-respiratory mycobacterial cultures performed per patient and limiting routine non-respiratory mycobacterial culture to only patients with risk factors for mycobacterial infection could reduce laboratory workload without substantially impacting patient care. Although the exact number of allowable cultures from patients with risk factors is ultimately at the discretion of the performing laboratory, employing a two-culture limit per patient would still allow for detection of > 90% of cases per our modeling analysis and could easily be incorporated into clinical decision support tools (CDSTs). For patients without risk factors, non-respiratory culture could be confined to laboratory director approval, depending on the volume of requests received; soft stops through CDST could also be considered.

Some laboratories, including our own, have attempted to control non-respiratory specimen volume by implementing protocols to combine specimens obtained from the same anatomic site before processing for mycobacterial smear and culture. While this approach does not require implementation of CDSTs, it has several disadvantages. First, laboratory personnel must take time to examine source information and then combine the appropriate specimens before proceeding with their workflow. Second, combining specimens with differing concentrations of mycobacteria and contaminating microbes can reduce sensitivity and increase the risk of non-mycobacterial contamination. Finally, clinicians might submit multiple specimens from sites that are distinct enough to fall outside the laboratory’s criteria for pooling. While potentially more difficult to implement, placing limits on non-respiratory specimens avoids these disadvantages and encourages clinicians to obtain the best specimen possible for testing.

Our study has limitations. First, it includes results from a single clinical laboratory in an area with low incidence of mycobacterial infection. Differences in swab types, specimen processing techniques, patient demographics, and prevalence of risk factors for mycobacterial infection at other institutions could limit the generalizability of these conclusions. Second, while digestion and decontamination are universally performed for swab specimens sent for mycobacterial culture in our laboratory, processing procedures vary among non-swab specimen types, which could affect mycobacterial culture-positive rates. Third, we based the classification of specimens on automated keyword searches in the specimen descriptions and result text; some specimens might have been misclassified in error. Fourth, we did not compare specimen source distributions across all specimens, and detailed chart review was limited to select positive results as it was not feasible to chart review every patient with a positive test. Finally, we have not yet evaluated the effects of the recommendations presented here on test utilization, positive rates, and laboratory workload.

In conclusion, our results inform practical strategies for improving fungal and mycobacterial test utilization. Such strategies will help clinical microbiology laboratories optimize management of limited personnel, financial resources, and space while supporting high-quality patient care.

## SUPPLEMENTAL MATERIAL

Supplemental material jcm.00030-26-s0001.docx Supplemental methods and results.

ASM does not own the copyrights to Supplemental Material that may be linked to, or accessed through, an article. The authors have granted ASM a non-exclusive, world-wide license to publish the Supplemental Material files. Please contact the corresponding author directly for reuse.
