# Supplementary material for: A myco-management problem: improving utilization of fungal and mycobacterial smear and culture
Source: J Clin Microbiol. 2026 Jun 9;64(7):e00030-26. doi: 10.1128/jcm.00030-26 (PMC13343888; doi:10.1128/jcm.00030-26)
Supplement: Supplemental material — Supplemental methods and results. [file jcm.00030-26-s0001.docx]

**SUPPLEMENTAL MATERIALS**

**Article title**

A Myco-Management Problem: Improving Utilization of Fungal and Mycobacterial Smear and Culture

**Authors**

Sarah Schrader, Vamsi Thiriveedhi, John Branda, Sarah Turbett, and Erik Klontz

Contents

[1. Risk factors for fungal and mycobacterial infection 2](#_Toc223864681)

[2. Mycobacterial smear and culture methods 3](#_Toc223864682)

[3. Fungal smear and culture methods 3](#_Toc223864683)

[4. Key words used for specimen classification 4](#_Toc223864684)

[5. Criteria used to determine clinical utility of results 15](#_Toc223864685)

[6. Detailed methods for estimation of the clinical effect of different non-respiratory specimen limits for mycobacterial culture 16](#_Toc223864686)

[7. Detailed methods for estimation of the laboratory workload reduction under different non-respiratory specimen limits for mycobacterial smear and culture 17](#_Toc223864687)

[6. TABLE S1: Cases in which a positive fungal smear performed on a swab-collected specimen affected clinical management. 21](#_Toc223864688)

[7. TABLE S2: Cases in which a positive mycobacterial smear performed on a swab-collected specimen affected clinical management. 22](#_Toc223864689)

[8. TABLE S3: Cases in which a positive non-respiratory mycobacterial smear affected clinical management. 23](#_Toc223864690)

[9. Supplemental references 24](#_Toc223864691)

# 1. Risk factors for fungal and mycobacterial infection

*Risk factors for fungal infection*

The Infectious Disease Society of America (IDSA) defines risk factors for invasive candidiasis (1) as:

- Candida colonization
- Severe illness requiring intensive level of care
- Broad-spectrum antibiotic exposure
- Recent major surgery
- Necrotizing pancreatitis
- Dialysis
- Parenteral nutrition
- Corticosteroid use
- Presence of a central venous catheter (CVC)

The IDSA defines risk factors for invasive aspergillosis (2) as:

- Prolonged neutropenia
- Allogeneic hematopoietic stem cell or solid organ transplant
- Corticosteroid use or infliximab therapy
- Advanced AIDS
- Chronic granulomatous disease

*Risk factors for mycobacterial disease*

The Centers for Disease Control and Prevention (CDC) defines risk factors for tuberculosis (TB) (3) as:

- Close contact with a person with active TB
- Immigration from an area with high TB rates
- Age less than five years with a positive TB test
- Membership in a group with a high rate of TB transmission (unhoused persons, persons who inject drugs, persons with HIV infection)
- Working or living with people at high risk for TB or in institutional settings

In addition, vaccination or intravesicular immunotherapy with BCG confers a small but well-documented risk of disseminated infection (4).

Recognized risk factors for nontuberculous mycobacterial infections (5–8) include:

- Chronic lung disease, including cystic fibrosis
- Allogeneic hematopoietic stem cell or solid organ transplant
- Corticosteroid use, infliximab therapy, or TNF-alpha therapy
- Advanced AIDS
- Open wound in contact with soil or water
- Cosmetic procedures involving contaminated equipment

# 2. Mycobacterial smear and culture methods

Mycobacterial smears were performed prior to digestion and decontamination for all specimens except sputum and bronchoalveolar lavage specimens. Heat-fixed slides were stained with auramine O with potassium permanganate counterstain and observed under a fluorescence microscope. Positive slides were graded as “rare (1+)”, “few (2+)”, or “abundant (3+ to 4+)”. Mycobacterial smears were not performed on urine specimens.

To prepare specimens for culture, solid tissue specimens were ground using a mortar & pestle or tissue grinder. Specimens from normally sterile sites, including bone marrow, cerebrospinal fluid (CSF), body fluids (unless cloudy-appearing), and surgically collected tissue specimens, were inoculated directly onto solid culture medium (Lowenstein Jensen slant with an additional chocolate agar slant for specimens from bone marrow, pericardial fluid, lymph nodes, ulcers, skin, or extremities or when the ordering clinician indicated suspicion for *Mycobacterium haemophilum*, *Mycobacterium marinum*, *Mycobacterium fortuitum*, *Mycobacterium chelonae*, or *Mycobacterium abscessus*) and into liquid culture medium. Nonsterile specimens, including respiratory fluids, skin specimens, gastric lavage fluid, urine, abscesses, and specimens obviously purulent or contaminated with non-mycobacterial flora, were digested and decontaminated with N-acetyl-L-cysteine and NaOH before inoculating onto solid and liquid media for mycobacterial culture. Respiratory tract specimens from cystic fibrosis patients were additionally treated with oxalic acid before inoculation. Cultures were incubated at 37°C except chocolate agar slants, which were incubated at 30°C. Additional liquid culture medium was inoculated and incubated at 42°C if the ordering clinician indicated suspicion for *Mycobacterium xenopi* or at 30°C if the ordering clinician indicated suspicion for *M. haemophilum*, *M. marinum*, *M. fortuitum*, *M. chelonae*, or *M. abscessus* or if the specimen was from an ulcer, skin, or extremity. Before finalizing the culture as negative, solid medium was routinely held for eight weeks and liquid medium for six weeks. For positive cultures, in-house organism identification was performed using MALDI-TOF mass spectrometry. For some isolates, identification was performed at reference laboratories using a combination of MALDI-TOF mass spectrometry and sequencing. Mycobacterial cultures were not performed on stool specimens.

# 3. Fungal smear and culture methods

Specimens received for both fungal smear and culture were generally used to inoculate solid medium before smear was performed. Solid tissue specimens were ground (or separated into two portions with one portion ground and the other minced when culture for zygomycetes was requested) prior to inoculation.

Smear was performed by staining specimens with calcofluor white and examining under an epifluorescence microscope. Positive results reflected the morphology of the fungal elements observed, recorded as: “budding yeast”, “yeast”, “round yeast”, “encapsulated yeast”, “hyphae,septated”, “hyphae,nonseptated”, and/or “fungal elements (yeast or mold)”. For positive cultures, in-house organism identification was performed using MALDI-TOF mass spectrometry for yeasts and on the basis of morphology for molds.

# 4. Key words used for specimen classification

When placing microbiology orders, the ordering clinician must indicate a specimen source from a fixed list and has the option to enter additional information in a free text “specimen information” field. Ordering clinicians can also enter special requests for the laboratory when placing an order. The dataset exported from the electronic health record (EHR) (Epic from Epic Systems, Verona, WI, USA) had separate columns for “result text” (containing special requests—which included both text from the providers and laboratory-entered specimen notes—and smear and/or culture result, with both results appearing in the same column if both were performed on a specimen), “source”, and “specimen information”. These columns were searched using the key words described below to classify specimens in an automated fashion using Python scripts. All key word searches were performed in a case-insensitive manner. Multiple manual checks were incorporated during development of each key word list to ensure accurate classification.

In some cases, a component of the result text was truncated due to immutable character limits imposed when exporting data from the EHR. To ensure accurate classification of affected specimens, we exported a complementary dataset from the laboratory information system (LIS) (Sunquest from Sunquest Information Systems, Tuscon, AZ, USA). Due to automatic filtering parameters, this dataset included most but not all specimens represented in the EHR dataset. This complementary dataset was used for automated classification of a small number of specimens in some cases as described below. A very small number of specimens that could not be classified by the automated algorithm were classified manually.

1. *Key words used to classify specimens as vaginal vs non-vaginal*

A specimen was classified as vaginal if the “source” or “specimen information” fields contained any of the following strings (some strings are purposely incomplete to capture multiple variations of the word):

- Vag
- Cervix
- Endocervi
- Vulva
- Vulvo

A specimen was also classified as vaginal if the source was “cervical” (this source choice refers to the cervix).

All other specimens were classified as non-vaginal.

1. *Key words used to classify specimens as respiratory vs non-respiratory*

We included as respiratory specimens sputa, bronchial alveolar lavage (BAL) fluid, tracheal aspirates, pleural fluid, chest tube drainage, throat swabs from cystic fibrosis patients, and non-lymph node tissue specimens from the lungs, bronchi, pleura, or trachea. In terms of key word searches, a specimen was classified as respiratory if the “source” field contained any of the following strings and the “specimen information” field did not contain “node” (to filter out lymph node specimens):

- BAL
- Bronchial
- Sputum
- Tracheal aspirate
- Endotracheal
- Pleural
- Chest tube
- Bronchus
- Lung
- Thf (abbreviation for thoracotomy fluid)

Because ordering clinicians sometimes used non-specific source categories for respiratory specimens (e.g., “tissue”, “wound”, “fluid”, etc.), it was also necessary to search the “specimen information” field. A specimen was classified as respiratory if the “specimen information” field contained any of the following strings and did not contain the string “node” (to filter out lymph nodes) or any of the other strings listed below as exceptions for certain entries:

- BAL (abbreviation for bronchoalveolar lavage)
  - Exceptions (other words containing the string “bal”): “ball”, “balan” (as in “balanitis”, “balanoposthitis”, etc.), “acetabalum” (misspelling of “acetabulum”), “vaginbal” (misspelling of “vaginal”)
- Bronch
- Sputum
- Trach or treach (misspelling of “trach”)
  - Exceptions: “paratracheal”
- Pleura
- Lung
- Right upper lobe or RUL
  - Exceptions (other words containing the string “rul”): “purulen” (as in “purulence” and “purulent”), “rule” (as in “rule out”), “rul…” (truncation of “rule out”)
- Right middle lobe or RML
- Right lower lobe or RLL
- Left upper lobe or LUL
  - Exceptions (other words containing the string “lul”): “intracellular”, “mandiblular” (misspelling of “mandibular”), “cellulitis”
- Left lower lobe or LLL
  - Exceptions (other words containing the string “lll”): “colllected” (misspelling of “collected”)
- Pulmonary
  - Exceptions: “aortic” (to filter out “aortic pulmonary window”), “valve” (to filter out “pulmonary valve”), “artery” (to filter out “pulmonary artery”)
- Lingula
- Chest tube
- Apical

A specimen was also classified as respiratory if the source was “tubing” and the string “chest” was present in the “specimen information” field or if the source was “throat” or “oral*” and the “specimen information” field contained “cystic fibrosis” or “cf” (to capture sputum specimens from cystic fibrosis patients entered under those source types).

In rare cases, the “source” and “specimen information” fields were too nonspecific to determine the nature of the specimen, but the specimen type was indicated in the “special requests” field. If “lung” was present in the result text (which contains the “special requests” component of the order) and the specimen type was not obviously non-respiratory (e.g., urine), the specimen was classified as respiratory.

All other specimens were classified as non-respiratory.

1. *Key words used to classify specimens as swab vs non-swab*

All specimens classified as “vaginal” were classified as swabs.

Other specimens were classified as swabs if the result text contained any of the following strings (part of specific text that is entered under the “special requests” component by the microbiology receiving desk upon receipt of a swab-collected specimen):

- Swab
- Culturette

In cases where the “special requests” component of the result text was truncated, the complementary LIS dataset was queried for the codes input by the microbiology receiving desk that indicate the specimen was received on a swab. For the small number of specimens with truncated “special requests” components that were not found in the LIS dataset, classification was performed manually.

1. *Key words used to classify specimens as from the OR vs not from the OR*

A specimen was classified as being received from the OR if the result text any of the following strings (part of specific text that is entered under the “special requests” component by the microbiology receiving desk upon receipt of a specimen from the OR):

- From OR
- From O.R.
- Surgical no (abbreviation for “surgical number”)

In addition, if the word “donor” was present in the “specimen information” field for a specimen classified as respiratory, the specimen was considered to represent a sample from a donor lung and was thus classified as having been collected in the OR.

In cases where the “special requests” component of the result text was truncated, the complementary LIS dataset was queried for the codes input by the microbiology receiving desk that indicate the specimen was received from the OR. For the small number of specimens with truncated “special requests” components that were not found in the LIS dataset, classification was performed manually.

1. *Key words used to classify smear results as positive vs negative*

For mycobacterial smear, the smear result was classified as positive if the result text contained any of the following strings uniquely associated with a positive mycobacterial smear result:

- Abundant
- Few
- Rare

If the result text contained “no acid fast bacilli”, it was classified as negative. For specimens for which the result text contained key words for both a positive and negative smear result (often due to a corrected result), classification was performed manually.

For fungal smear, the smear result was classified as positive if the result text contained any of the following strings uniquely associated with a positive fungal smear result and the string was not preceded by “previously reported as: “ (which would indicate a corrected result). Some of the strings (*e.g.*, “yeast”) included the header that preceded the fungal smear result (“fungal wet prep: “) because the string could appear in other contexts, while the header was omitted for others (*e.g*., “budding yeast”) because the string only appeared in the context of a positive fungal smear result.

- Budding yeast
- Fungal wet prep: yeast
- Fungal wet prep: round
- Fungal wet prep: encapsulated yeast
- Hyphae,septated
- Hyphae,nonseptated
- Fungal elements (yeast or mold)

If the result text contained “no fungi seen” (the standard text for a negative fungal smear result) or one of the two non-standard results “fungal wet prep: no fungus or yeast isolated after 28 days” and “fungal wet prep: no growth 28 days” (likely reflective of coding errors where the code for a negative culture was mistakenly used instead of the code for a negative smear), it was classified as negative.

For both mycobacterial and fungal smear, if the header preceding the smear result (“acid fast stain: ” for mycobacterial smear and “fungal wet prep: ” for fungal smear) was truncated, the complementary LIS dataset was queried for the codes input by the microbiology staff that indicated a positive or negative result. For the small number of specimens that were not found in the laboratory information system dataset, classification was performed manually.

1. *Key words used to classify culture results as positive vs negative*

The result text—which included both smear and culture results if both were performed on the same specimen—was first split on the fixed headers “fungal/yeast culture: ” (for fungal culture) or “mycobacterium culture: ” (for mycobacterial culture) to isolate the culture result. For fungal culture, if the text after the header for a specimen contained “no fungus or yeast isolated”, “no fungus isolated”, or “no growth”, the culture result was classified as negative. If the culture had not already been classified as negative and the text after the header contained any of the following strings, the culture result was classified as positive.

- Genus names:
  - *Acremonium*
  - *Aspergillus*
  - *Candida*
  - *Cryptococcus*
  - *Fonsecaea*
  - *Fusarium*
  - *Geotrichum*
  - *Gordonia*
  - *Graphium*
  - *Mucor*
  - *Mycobacterium*
  - *Nocardia*
  - *Paecilomyces*
  - *Penicillum*
  - *Phyton* (common component of *Trichophyton* and *Epidermophyton*)
  - *Pithomyces*
  - *Rhodococcus*
  - *Rhodotorula*
  - *Saccharomyces*
  - *Saprochaete*
  - Scedosporium
  - *Tricho* (common component of *Trichosporon*, *Trichophyton*, and *Trichoderma*)
  - *Tsukamurella*
- General words associated with organism identifications:
  - Acid fast bacilli
  - Colony (as in “one colony of…”)
  - Colonies (as in “two colonies of…”)
  - Complex (included in complex-level identifications)
  - Fungi
  - Modified acid fast bacilli
  - Mold
  - Saprophytic
  - Species (added for genus-level identifications)
  - Yeast
- Other key words always indicative of a positive culture:
  - Similar growth (used for mycobacterial culture to refer to a previous culture performed within three months that is growing an organism with the same morphology)
  - See bacterial culture (used to refer to a concurrent bacterial culture growing the same organism)
  - Identification performed (used when identification is performed by a reference laboratory)

For mycobacterial culture specimens that had not already been classified as positive, if the text after the header for a specimen contained “negative for mycobacteria”, the culture result was classified as negative (this was performed after identification of positive cultures because if a modified acid-fast organism grew, the culture result sometimes still included “negative for mycobacteria”).

For specimens for which the “special requests” component of the result text was truncated, the entire result text was searched for specific strings always associated with a positive or negative result. For fungal culture, if the result text contained “no fungus or yeast isolated after” (which is followed by a variable number of days for which the culture was incubated), it was classified as negative. If the result text contained “probable dermatophyte” or “which failed to grow on subculture”, it was classified as positive. For mycobacterial culture, if the result text contained “mycobacterium culture: result called to care unit and/or md” or “mycobacterium culture: md and/or care unit notified” and one of the following strings, it was classified as positive:

- Similar growth
- Identification performed
- See bacterial culture
- Which failed to grow on subculture
- Isolate is being sent to a reference lab
- See later culture for sensitivities
- See previous susceptibility testing

For the small number of specimens with truncated culture result text components that could not be classified by the above algorithm, classification was performed manually.

1. *Key words used to classify fungal smears by result type (yeast vs mold)*

For specimens with positive fungal smear results, the smear result was isolated from the rest of the result text by splitting the result text on all variations of the fungal smear header (full and truncated forms) and the fungal culture header (full and truncated forms). Isolated smear results containing the “yeast” outside the context of “fungal elements (yeast or mold)” were classified as yeast, results containing “hyphae,septated” or “hyphae,nonseptated” were classified as mold, and results containing “fungal elements” were classified as unspecified fungal elements. For specimens with multiple fungal smear results (*e.g.*, both yeast and true hyphae reported), each result was classified separately and then combined into a list to reflect all the fungal morphologies identified in the smear.

1. *Key words used to classify fungal cultures by result type (yeast vs mold)*

For specimens with positive fungal culture results, the culture result was isolated from the rest of the result text by splitting the result text on all variations of the fungal culture header (full and truncated forms). Text not part of an organism name, including qualifiers (*e.g.*, “one colony of”, “few”, “rare”) and laboratory comments (*e.g.*, regarding result notification, susceptibility results, etc.), was removed to isolate the organism name to a reasonable degree. A list of unique organism names was exported for manual classification as yeast, mold, dimorphic, or actinomycete (see table below). The organism names cultured from each specimen were then referenced against this list to classify the result as yeast or mold. For specimens with multiple organisms isolated from culture, each organism was classified separately and then combined into a list to reflect all the organism types cultured from the specimen.

| Culture result | Classification |
| --- | --- |
| acid fast bacilli isolated | actinomycete |
| acremonium species | mold |
| acrodontium salmoneum | mold |
| akanthomyces species | mold |
| alternaria species | mold |
| arthrographis | mold |
| aspergillus flavus | mold |
| aspergillus fumigatus | mold |
| aspergillus nidulans | mold |
| aspergillus niger | mold |
| aspergillus species | mold |
| aspergillus species : aspergillus calidoustus | mold |
| aspergillus species (aspergillus jensenii) | mold |
| aspergillus species (aspergillus ochroaceus) | mold |
| aspergillus species (closest identity to aspergillus udagawae, a member of viridinutans c... | mold |
| aspergillus terreus | mold |
| aspergillus versicolor | mold |
| aureobasidium melanogenum | yeast |
| aureobasidium species | yeast |
| beauveria sp | mold |
| blastomyces dermatitidis | dimorphic |
| candida albicans | yeast |
| candida albicans and candida krusei | yeast |
| candida auris | yeast |
| candida dubliniensis | yeast |
| candida famata | yeast |
| candida glabrata | yeast |
| candida guilliermondii | yeast |
| candida haemulonii | yeast |
| candida inconspicua | yeast |
| candida intermedia | yeast |
| candida kefyr (pseudotropicalis) | yeast |
| candida krusei | yeast |
| candida lambica | yeast |
| candida lipolytica | yeast |
| candida lusitaniae | yeast |
| candida metapsilosis | yeast |
| candida norvegensis | yeast |
| candida orthopsilosis | yeast |
| candida parapsilosis | yeast |
| candida pelliculosa | yeast |
| candida rugosa | yeast |
| candida species | yeast |
| candida species (candida parapsilosis complex) | yeast |
| candida species, not albicans | yeast |
| candida tropicalis | yeast |
| candida zeylanoides | yeast |
| chaetomium species | mold |
| chrysosporium | mold |
| cladophialophora bantiana | mold |
| cladosporium species | mold |
| cladosporium species (cladosporium funiculosum) | mold |
| coccidioides species | dimorphic |
| coccidioides species (coccidioides posadasii) | dimorphic |
| coccidioides species (coccidioides posadasii) | dimorphic |
| cryptococcus albidus | yeast |
| cryptococcus laurentii | yeast |
| cryptococcus neoformans | yeast |
| cryptococcus terreus | yeast |
| dematiaceous mold | mold |
| dematiaceous mold :(aureobasidium melanogenum) | mold |
| dematiaceous mold (exophiala oligosperma) | mold |
| dematiaceous mold (possible curvularia sp) | mold |
| dematiaceous mold (probable cladosporium halotolerans) | mold |
| dematiaceous mold (pseudopithomyces palmicola) | mold |
| dematiaceous mold cladophialophora bantiana | mold |
| dermatophyte | mold |
| epidermophyton floccosum | yeast |
| exophiala (wangiella) dermatitidis | yeast |
| exophiala species : exophiala oligosperma | yeast |
| fonsecaea pedrosoi | mold |
| fungi | mold |
| fungi (rasamsonia piperina) | mold |
| fungi (acremonium egyptiacum) | mold |
| fungi (acrodontium salmoneum) | mold |
| fungi (arthrogaphis kalrae) | mold |
| fungi (arthrographis kalrae) | mold |
| fungi (arthrographis multiformispora) | mold |
| fungi (blastobotrys aristatus) | mold |
| fungi (hydnophlebia chrysorhiza) | mold |
| fungi (hypoxylon crocopeplum) | mold |
| fungi (irpex lacteus) | mold |
| fungi (microascus gracilis) | mold |
| fungi (mycoacia fuscoatra) | mold |
| fungi (paraphaeosphaeria species) | mold |
| fungi (phaeoacremonium scolyti) | mold |
| fungi (phlebia species) | mold |
| fungi (probable arthrographis) | mold |
| fungi (probable cadophora malorum) | mold |
| fungi (probable corynascella humicola) | mold |
| fungi (thyridium species) | mold |
| fungi (trichophyton violaceum) | mold |
| fungi (valsonectria roseola formerly acremonium roseolum) | mold |
| fungi parengyodontium album | mold |
| fungi purpureocillium lilacinum formerly paecilomyces lilacinus | mold |
| fusarium species | mold |
| fusarium species (fusarium keratoplasticum) | mold |
| fusarium species (fusarium proliferatum) | mold |
| fusarium species (fusarium solani) | mold |
| gamszarea testudinea | mold |
| geotrichum capitatum | yeast |
| geotrichum species | yeast |
| gloeophyllum sepiarium | mold |
| gordonia bronchialis | actinomycete |
| graphium | mold |
| histoplasma capsulatum | dimorphic |
| histoplasma suramericanum ( histoplasma capsulatum ) | dimorphic |
| kodamaea (pichia) ohmeri | yeast |
| lichtheimia (absidia/mycocladus) species | mold |
| malassezia pachydermatis | yeast |
| malassezia species | yeast |
| malbranchea species | mold |
| microascus cirrosus | mold |
| microsporum species | mold |
| modified acid fast bacilli isolated | actinomycete |
| mold | mold |
| mucor species | mold |
| mucor species (mucor circinelloides) | mold |
| mucor species (mucor lusitanicus) | mold |
| mucor species (mucor pseudolusitanicus) | mold |
| mycobacterium abscessus | actinomycete |
| mycobacterium abscessus complex | actinomycete |
| mycobacterium abscessus subsp abscessus | actinomycete |
| mycobacterium chelonae | actinomycete |
| nocardia nova complex | actinomycete |
| nocardia species arthritidis | actinomycete |
| nocardia transvalensis complex | actinomycete |
| ochroconis gallopava (dactylaria constricta) | mold |
| paecilomyces lilacinus (purpureocillium lavendulum) | mold |
| paecilomyces lilacinus (purpureocillium lilacinum) | mold |
| paecilomyces species | mold |
| penicillium species | mold |
| penicillium species :(penicillium cluniae) | mold |
| penicillium species :talaromyces (penicillium) acaricola | mold |
| peniophora species (russulales) | mold |
| presumptive coccidioides species | dimorphic |
| probable arthrographis | mold |
| probable acremonium species | mold |
| probable aspergillus fumigatus | mold |
| probable candida metapsilosis | yeast |
| probable candida species c. metapsilosis | yeast |
| probable candida species c. orthopsilosis | yeast |
| probable coccidioides species | dimorphic |
| probable coniochaeta deborreae | mold |
| probable cryptococcus terreus | yeast |
| probable cryptococcus uniguttulatus | yeast |
| probable dermatophyte | mold |
| probable lodderomyces elongisporus | yeast |
| probable rhodotorula species | yeast |
| pseudallescheria boydii complex | mold |
| rhizopus species | mold |
| rhizopus species : rhizopus microsporus | mold |
| rhizopus species (rhizopus arrhizus var. arrhizus) | mold |
| rhodotorula minuta | yeast |
| rhodotorula mucilaginosa | yeast |
| rhodotorula species | yeast |
| rigidoporus corticola (formerly oxyporus corticola) | mold |
| saccharomyces cerevisiae | yeast |
| saccharomyces species | yeast |
| saprochaete capitata (formerly geotrichum capitatum) | yeast |
| saprophytic fungus | mold |
| saprophytic fungus : rigidoporus corticola | mold |
| scedosporium apiospermum/pseudallescheria boydii | mold |
| scedosporium prolificans | mold |
| scopulariopsis species | mold |
| scytalidium species | mold |
| syncephalastrum species | mold |
| trichoderma | mold |
| trichophyton rubrum | mold |
| trichophyton species | mold |
| trichophyton species :(trichophyton violaceum) | mold |
| trichophyton tonsurans | mold |
| trichophyton violaceum | mold |
| trichosporon asahii | yeast |
| trichosporon inkin | yeast |
| trichosporon mucoides | yeast |
| trichosporon species | yeast |
| tsukamurella tyrosinosolvens | actinomycete |
| valsonectria roseola (formerly acremonium roseolum) | mold |
| verticillium species | mold |
| yeast | yeast |
| yeast papiliotrema fonsecae (formerly cryptococcus fonsecae) | yeast |
| yeast (brettanomyces bruxellensis) | yeast |
| yeast (not candida albicans) | yeast |
| yeast (not candida albicans) (cystobasidium minutum) | yeast |
| yeast (probable cryptococcus sp., not cryptococcus neoformans) | yeast |
| yeast candida albicans | yeast |
| yeast probable cystobasidium sp | yeast |

# 5. Criteria used to determine clinical utility of results

For positive smears from swabs, results were considered clinically impactful if:

1. No concurrent non-swab specimen had a smear or Gram stain with the same result,
2. The result was considered to represent a true pathogen based on documentation in the clinical notes, and
3. The result prompted a change in clinical management, defined as changes to antimicrobial therapy, surgical intervention, or other substantial alteration to clinical care such as reduction of immunosuppressive medication dose.

If two concurrent swabs both had positive smears and the above criteria were met, only one was counted as having had an impact on clinical management.

For patients with at least one positive non-respiratory mycobacterial smear and/or culture in 2021-2022, we determined:

1. The number and result of mycobacterial smears and cultures performed on non-respiratory specimens during the two weeks before and two weeks after the first positive non-respiratory specimen,
2. Whether the organism grew in a concurrent non-mycobacterial culture,
3. Whether the result was considered to represent a true pathogen based on documentation in the clinical notes,
4. Whether the diagnosis had already been established at the time of the result,
5. Whether the patient had risk factors for mycobacterial infection, and
6. Whether the smear result had changed clinical management as described above (for smear-positive specimens).

# 6. Detailed methods for estimation of the clinical effect of different non-respiratory specimen limits for mycobacterial culture

For each of the 29 cases from 2021-2022 in which mycobacterial culture provided a microbiologic diagnosis, we used the total number of non-respiratory mycobacterial cultures performed during the two weeks before and two weeks after the first positive mycobacterial culture along with the total number of positive cultures in that period to calculate the probability that the patient would have had at least one positive culture under specimen limits of one, two, or three.

If the total number of cultures was less than or equal to the limit, then the probability of a positive culture was 1.

If the total number of cultures was greater than the limit, we first determined the number of unique culture combinations possible given each limit using the formula for determining the number of combinations of size *r* from a set of size *n*, where *r* represents the hypothetical culture limit and *n* represents the number of cultures the patient had performed:

$$C_{r}^{n}=\frac{n!}{r!\left( n-r \right)!}$$

We then determined the number of those unique combinations that would contain one or more positive cultures. To do so, we calculated the number of unique combinations that would contain only negative cultures and then subtracted it from the total (the same formula as above can be used, with *r* representing the hypothetical culture limit and *n* representing the number of negative cultures). The probability of at least positive culture (*P*) was then:

$$P=\frac{number of combinations of size r with \geq1 positive culture}{total number of combinations of size r}$$

For example, for a patient with three cultures (A, B, and C), one of which was positive (A), the calculations would be as follows:

For a limit of one culture:

$$C_{1}^{3}=\frac{3!}{1!\left( 3-1 \right)!}=\frac{3*2*1}{1*(2*1)}= \frac{6}{2}=3$$

Thus, there are three unique combinations of one culture possible (A, B, or C). Because only one culture was positive, only one of these combinations (A) can have at least one positive culture (two—B and C—will contain only negative cultures). The probability of at least one positive culture is then:

$$P=\frac{1}{3}=\sim0.333$$

For a limit of two cultures:

$$C_{2}^{3}=\frac{3!}{2!\left( 3-2 \right)!}=\frac{3*2*1}{2*1*(1)}= \frac{6}{2}=3$$

Thus, there are three unique combinations of two cultures possible (A & B, A & C, and B & C). One (B & C) contains only negative cultures, so two contain at least one positive culture. The probability of at least one positive culture is then:

$$P=\frac{2}{3}=\sim0.667$$

For a limit of three cultures:

$$C_{3}^{3}=\frac{3!}{3!\left( 3-3 \right)!}=\frac{3*2*1}{3*2*1*(1)}= \frac{6}{6}=1$$

Thus, there is one unique combination of three cultures possible (A & B & C). This combination contains at least one positive culture. The probability of at least one positive culture is then:

$$P=\frac{3}{3}=1$$

#

The numbers used to make these calculations for each of the 29 cases are summarized in the table below:

| Case(s) | Number of non-resp. cultures | Number of positive cultures | Limit one culture | | | Limit two cultures | | | Limit three cultures | | |
| --- | --- | --- | --- | --- | --- | --- | --- | --- | --- | --- | --- |
|  |  |  | Total unique combos | Combos with ≥ 1 positive | *P* of ≥ 1 positive | Total unique combos | Combos with ≥ 1 positive | *P* of ≥ 1 positive | Total unique combos | Combos with ≥ 1 positive | *P* of ≥ 1 positive |
| 1-13 | 1 | 1 | 1 | 1 | 1 | 1 | 1 | 1 | 1 | 1 | 1 |
| 14-15 | 2 | 1 | 2 | 1 | 0.5 | 1 | 1 | 1 | 1 | 1 | 1 |
| 16-18 | 2 | 2 | 2 | 2 | 1 | 1 | 1 | 1 | 1 | 1 | 1 |
| 19-20 | 3 | 1 | 3 | 1 | 0.33 | 3 | 2 | 0.67 | 1 | 1 | 1 |
| 21 | 3 | 2 | 3 | 2 | 0.67 | 3 | 3 | 1 | 1 | 1 | 1 |
| 22 | 3 | 3 | 3 | 3 | 1 | 3 | 3 | 1 | 1 | 1 | 1 |
| 23-24 | 4 | 1 | 4 | 1 | 0.25 | 6 | 3 | 0.5 | 4 | 3 | 0.75 |
| 25 | 4 | 2 | 4 | 2 | 0.5 | 6 | 5 | 0.83 | 4 | 4 | 1 |
| 26-28 | 5 | 2 | 5 | 2 | 0.4 | 10 | 7 | 0.7 | 10 | 9 | 0.9 |
| 29 | 7 | 5 | 7 | 5 | 0.71 | 21 | 20 | 0.95 | 35 | 35 | 1 |

# 7. Detailed methods for estimation of the laboratory workload reduction under different non-respiratory specimen limits for mycobacterial smear and culture

To estimate the reduction in laboratory workload under different non-respiratory specimen limits for mycobacterial smear (0, 1, or 2) and culture (1, 2, or 3), we determined the following:

1. The total number of mycobacterial smears and total number of mycobacterial cultures performed by our laboratory from January 1^st^, 2021, to December 31^st^, 2022.
2. The number of those smears/cultures that were performed on non-respiratory specimens.
3. For each test, the number of unique patients who had at least one non-respiratory specimen processed.
4. For smears, the number of unique patients who had one non-respiratory smear and the number who had at least two non-respiratory smears.
5. For cultures, the number of unique patients who had one non-respiratory culture, the number who had two non-respiratory cultures, and the number who had at least three non-respiratory cultures.

Those numbers are as follows:

| Parameter | Smear | Culture |
| --- | --- | --- |
| Total volume | 20,975 | 20,851 |
| Non-respiratory volume | 11,030 | 11,057 |
| Number of unique patients (non-respiratory) | 4,497 | 4,539 |
| Number of unique patients with one non-respiratory test | 2,306 | 2,332 |
| Number of unique patients with two or more non-respiratory tests | 2,191 | - |
| Number of unique patients with two non-respiratory tests | - | 845 |
| Number of unique patients with three or more non-respiratory tests | - | 1,362 |

We then calculated the estimated workload reduction for each test under each hypothetical limit as follows:

*Mycobacterial smear*

- No non-respiratory smears:
  - The reduction in non-respiratory smear volume would be 11,030.
  - This represents a 11,030/20,975 x 100% = 52.6% reduction in total smear volume.
- Limit of one non-respiratory smear per patient:
  - All patients would be limited to one smear each = 4,497 non-respiratory smears.
  - The reduction in non-respiratory smear volume would thus be 11,030 – 4,497 = 6,533.
  - This represents a 6,533/20,975 x 100% = 31.1% reduction in total smear volume.
- Limit of two non-respiratory smears per patient:
  - The 2,306 patients with one non-respiratory smear would still have one smear each = 2,306 smears.
  - The 2,191 patients with at least two non-respiratory smears would be limited to two smears each = 4,382 smears.
  - The total number of non-respiratory smears would thus be 2,306 + 4,382 = 6,688.
  - The reduction in non-respiratory smear volume would thus be 11,030 – 6,746 = 4,342.
  - This represents a 4,342/20,975 x 100% = 20.7% reduction in total smear volume.

*Mycobacterial culture*

- Limit of one non-respiratory culture per patient:
  - All patients would be limited to one culture each = 4,539 non-respiratory cultures.
  - The reduction in non-respiratory culture volume would thus be 11,057 – 4,539 = 6,518.
  - This represents a 6,518/20,851 x 100% = 31.3% reduction in total culture volume.
- Limit of two non-respiratory cultures per patient:
  - The 2,332 patients with one non-respiratory culture would still have one culture each = 2,332 cultures.
  - The 845 + 1,362 = 2,207 patients with at least two non-respiratory cultures would be limited to two cultures each = 4,414 cultures.
  - The total number of non-respiratory cultures would thus be 2,332 + 4,414 = 6,746.
  - The reduction in non-respiratory culture volume would thus be 11,057 – 6,746 = 4,311.
  - This represents a 4,311/20,851 x 100% = 20.7% reduction in total culture volume.
- Limit of three non-respiratory culture per patient:
  - The 2,332 patients with one non-respiratory culture would still have one culture each = 2,332 cultures.
  - The 845 patients with two non-respiratory cultures would still have two cultures each = 1,690 cultures.
  - The 1,362 patients with three or more non-respiratory cultures would be limited to three cultures each = 4,086 cultures.
  - The total number of non-respiratory cultures would thus be 2,332 + 1,690 + 4,086 = 8,108.
  - The reduction in non-respiratory culture volume would thus be 11,057 – 8,108 = 2,949.
  - This represents a 2,949/20,851 x 100% = 14.1% reduction in total culture volume.

# 6. TABLE S1: Cases in which a positive fungal smear performed on a swab-collected specimen affected clinical management.

| Case | Patient summary | Swab specimen source | Swab smear result | Management change | Swab culture result | Time to next equivalent result (test)* |
| --- | --- | --- | --- | --- | --- | --- |
| 1 | Male in his teens with Crohn's disease who was admitted for planned ileocecal resection. Developed persistent post-operative pain with abdominal imaging concerning for intraabdominal infection due to bowel perforation or anastomotic leak. | peritoneal fluid | budding yeast | fluconazole started | Negative | N/A |
| 2 | Male in his 20s who was admitted with severe head injury requiring multiple cranial operations. Concern for surgical site infection at an area of dehiscence. | cranial wound | budding yeast | micafungin started | Negative | N/A |
| 3 | Female in her 50s who presented with an infected decubitus ulcer and perineal abscess requiring debridement. | perineal abscess | budding yeast | micafungin started | *Candida albicans* | 24 h (fungal culture from the same specimen) |
| 4 | Male in his 50s with cirrhosis who was admitted with abdominal pain secondary to colonic perforation. | peritoneal fluid | budding yeast | micafungin started | *Saccharomyces cerevisiae* | 6 h (fungal culture from the same specimen) |
| 5 | Female in her 70s with diabetes who was admitted with a diffuse, painful rash. | leg pustule | budding yeast with pseudohyphae | fluconazole started, betamethasone cream discontinued | *Candida albicans* | 26 h (fungal culture from a different specimen) |
| 6 | Female in her teens who was admitted with cardiac arrest secondary to diabetic ketoacidosis with hyperosmolar hyperglycemic syndrome. Course complicated by compartment syndrome requiring fasciotomy, which subsequently developed signs of infection. | hand, fasciotomy site | hyphae, septated | patient taken to OR for debridement | *Penicillium* spp. | 21 h (fungal culture from a different specimen) |

*“Time to next equivalent result (test)” indicates the time difference between the fungal smear result and the next result that provided equivalent information. The test that provided equivalent information is indicated in parenthesis.

# 7. TABLE S2: Cases in which a positive mycobacterial smear performed on a swab-collected specimen affected clinical management.

| Case | Patient summary | Specimen source | Smear result | Management change | Culture result | Time to next equivalent result (test)* |
| --- | --- | --- | --- | --- | --- | --- |
| 1 | Female in her 40s who was admitted for a surgical wound infection following cosmetic procedure in the Caribbean. | thigh wound | few (2+) acid-fast bacilli | amikacin, imipenem, linezolid, and azithromycin started | *Mycobacterium fortuitum* group | 5 days (mycobacterial culture from same specimen) |
| 2 | Male in his 70s who was admitted for left great toe infection. | toe | abundant (3 to 4+) acid-fast bacilli | empiric cefepime and metronidazole discontinued | *Mycobacterium chelonae* | 2 days (non-mycobacterial culture from a different specimen) |
| 3 | Male in his 50s with rheumatoid arthritis on immunosuppressants who was admitted for a non-healing surgical wound after hardware placement. | foot bone | rare (1+) acid-fast bacilli | immunosuppression reduced, patient admitted and taken to OR for repeat debridement | Negative | N/A - considered not to represent true infection after repeat procedure with negative smear and culture |

*“Time to next equivalent result (test)” indicates the time difference between the mycobacterial smear result and the next result that provided equivalent information. The test that provided equivalent information is indicated in parenthesis.

# 8. TABLE S3: Cases in which a positive non-respiratory mycobacterial smear affected clinical management.

| Case | Patient summary | Specimen source | Smear result | Management change | Culture result | Time to next equivalent result (test)* |
| --- | --- | --- | --- | --- | --- | --- |
| 1 | Male in his 30s with advanced HIV who was admitted with progressive fatigue and lymphadenopathy. | lymph node | abundant (3 to 4+) acid-fast bacilli | therapy targeting tuberculosis and atypical mycobacteria initiated | Negative (*Mycobacterium genavense* via molecular testing) | 2 days (anatomic pathology) |
| 2 | Female in her 40’s with cranial hardware who presented with cranial hardware/bone infection. | cranial wound (two specimens) | abundant (3 to 4+) acid-fast bacilli & rare (1+) acid-fast bacilli | therapy targeting a presumed slow-growing mycobacterial species initiated | Negative | N/A |
| 3 | Female in her 40s who was admitted for a surgical wound infection following cosmetic procedure in the Caribbean. | thigh wound | few (2+) acid-fast bacilli | amikacin, imipenem, linezolid, and azithromycin started | *Mycobacterium fortuitum* group | 5 days (mycobacterial culture from the same specimen) |
| 4 | Male in his 70’s from Vietnam with a history of positive purified protein derivative (PPD) who presented with progressive neck swelling. | lymph node (two specimens) | rare (1+) acid-fast bacilli (both specimens) | tuberculosis therapy initiated | *Mycobacterium tuberculosis* complex | 6 days (cytology) |
| 5 | Female in her 20s from India who presented with subacute altered mental status and headache concerning for tuberculous vs fungal meningitis. Started on empiric tuberculosis therapy and amphotericin B. | Cerebrospinal fluid | rare (1+) acid-fast bacilli | empiric amphotericin B discontinued | Negative (another CSF specimen grew *Mycobacterium tuberculosis* complex) | 15 days (mycobacterial culture from a different specimen) |

*“Time to next equivalent result (test)” indicates the time difference between the mycobacterial smear result and the next result that provided equivalent information. The test that provided equivalent information is indicated in parenthesis.

# 9. Supplemental references

1. Pappas PG, Kauffman CA, Andes DR, Clancy CJ, Marr KA, Ostrosky-Zeichner L, Reboli AC, Schuster MG, Vazquez JA, Walsh TJ, Zaoutis TE, Sobel JD. 2016. Clinical Practice Guideline for the Management of Candidiasis: 2016 Update by the Infectious Diseases Society of America. Clin Infect Dis 62:e1-50.

2. Patterson TF, Thompson GR, Denning DW, Fishman JA, Hadley S, Herbrecht R, Kontoyiannis DP, Marr KA, Morrison VA, Nguyen MH, Segal BH, Steinbach WJ, Stevens DA, Walsh TJ, Wingard JR, Young J-AH, Bennett JE. 2016. Practice Guidelines for the Diagnosis and Management of Aspergillosis: 2016 Update by the Infectious Diseases Society of America. Clinical Infectious Diseases 63:e1–e60.

3. CDC. 2025. Tuberculosis Risk Factors. Tuberculosis (TB). https://www.cdc.gov/tb/risk-factors/index.html. Retrieved 3 November 2025.

4. Lobo N, Brooks NA, Zlotta AR, Cirillo JD, Boorjian S, Black PC, Meeks JJ, Bivalacqua TJ, Gontero P, Steinberg GD, McConkey D, Babjuk M, Alfred Witjes J, Kamat AM. 2021. 100 years of Bacillus Calmette–Guérin immunotherapy: from cattle to COVID-19. Nat Rev Urol 18:611–622.

5. Loebinger MR, Quint JK, van der Laan R, Obradovic M, Chawla R, Kishore A, van Ingen J. 2023. Risk Factors for Nontuberculous Mycobacterial Pulmonary Disease: A Systematic Literature Review and Meta-Analysis. Chest 164:1115–1124.

6. Chung J, Ince D, Ford BA, Wanat KA. 2018. Cutaneous Infections Due to Nontuberculosis Mycobacterium: Recognition and Management. Am J Clin Dermatol 19:867–878.

7. Axson EL, Bual N, Bloom CI, Quint JK. 2019. Risk factors and secondary care utilisation in a primary care population with non-tuberculous mycobacterial disease in the UK. Eur J Clin Microbiol Infect Dis 38:117–124.

8. Honda JR, Alper S, Bai X, Chan ED. 2018. Acquired and genetic host susceptibility factors and microbial pathogenic factors that predispose to nontuberculous mycobacterial infections. Current Opinion in Immunology 54:66–73.
